# Boronic Acid Group: A Cumbersome False Negative Case in the Process of Drug Design

**DOI:** 10.3390/molecules21091185

**Published:** 2016-09-07

**Authors:** Sotirios Katsamakas, Anastasios G. Papadopoulos, Dimitra Hadjipavlou-Litina

**Affiliations:** 1 Department of Pharmaceutical Chemistry, School of Pharmacy, Aristotle University of Thessaloniki, University Campus, 54124 Thessaloniki, Greece; sotikats@pharm.auth.gr; 2 Laboratory of Applied Quantum Chemistry, School of Chemistry, Aristotle University of Thessaloniki, University Campus, 54124 Thessaloniki, Greece; anastp@chem.auth.gr

**Keywords:** virtual screening, autotaxin, boronic acid, organoboron, drug design, warhead

## Abstract

Herein we present, an exhaustive docking analysis considering the case of autotaxin (ATX). HA155, a small molecule inhibitor of ATX, is co-crystallized. In order to further extract conclusions on the nature of the bond formed between the ligands and the amino acid residues of the active site, density functional theory (DFT) calculations were undertaken. However, docking does not provide reproducible results when screening boronic acid derivatives and their binding orientations to protein drug targets. Based on natural bond orbital (NBO) calculations, the formed bond between Ser/Thr residues is characterized more accurately as a polar covalent bond instead of a simple nonpolar covalent one. The presented results are acceptable and could be used in screening as an active negative filter for boron compounds. The hydroxyl groups of amino acids are bonded with the inhibitor’s boron atom, converting its hybridization to sp^3^.

## 1. Introduction

Boronic acid is considered as a bioisostere for carboxylic acids bearing lower acidity [1]. It emerged as a pharmacophore group after the clinical approval of the drug bortezomib [2] in 2003 as an anticancer agent. Boron is located before carbon in the periodic table of elements. Both elements present similarities in behavior when participating in organic molecules [3]. In nature, boron-containing compounds can be found in plants, algae, microorganisms [4], and in certain natural products derived from bacteria. [5] Boron contains an empty *p* orbital in its sp^2^ hybridization state, presenting a trigonal planar geometry. Upon covalent bond formation, sp^3^ hybridization takes place, leading to a tetrahedral geometry [6].

Several recent reviews pointed to the significance of boron-containing compounds and to their medicinal interest. Boron derivatives were found to present various biological activities: Neuroprotection [7], antifungal [8], anti-inflammatory [9,10,11,12,13,14], anti-oxidant [15], anti-trypanosomial [16], antidiabetic [17], anticancer [2,9,10,11,12,18], antibacterial [19,20] and/or antiviral [21]. Recently, a second boron compound, tavaborole [8], gained approval as a topical antifungal agent. Thus, boronic acid derivatives continuously attract researchers’ attention for medicinal applicability as novel “lead compounds”. However, their biological mechanism of action and their mutagenic activity in mammals are still under study and are not well defined. Thus, several researchers have tested various boronic acid derivatives as mutagenic risk factors using the Ames assay as a reference protocol of mutagenicity. Bortezomib and the tested compounds were registered as Ames-negative [22].

Based on our previous 2D-QSAR and 3D in silico studies on boronic acid derivatives [23,24], we present herein a docking analysis in order to define the typical steps and variables that are important to incorporate in a library’s virtual screening when organoboron derivatives are included. The enzyme autotaxin (ATX) was used as a biological target in our investigation. The enzyme is located extracellularly and participates in lipid metabolism, converting lysophosphatidylcholine (LPC) to lysophosphatidic acid (LPA). Its active site is located within its catalytic phosphodiesterase (PDE) domain with known recorded XRD data (PDB entry *2XRG*) in the presence of a small molecule inhibitor HA155 [25], presenting a threonine-mediated covalent bond between the enzyme and the inhibitor. Moreover, we performed DFT calculations in order to explore the nature of the covalent bond formed between the inhibitor HA155 and two residues similar like Ser/Thr. Additionally, boron compounds are known to form covalent bonds with His [26] and/or Asp [18] residues. The use of covalent “warhead” groups in Medicinal Chemistry are an emerging strategy to achieve irreversible protein inhibition [27,28]. Hence, designed molecules bearing a covalent warhead can be used in targeting shallow pockets or clefts at the surface of proteins to selectively disrupt protein–protein interactions (PPIs) [29] in vivo and in other contexts.

## 2. Results and Discussion

**Scheme 1:**
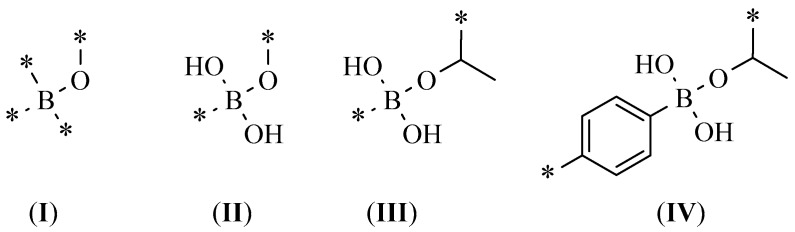
General smart pattern representation structures (**I, II, III, IV**) used for the performed constrained docking simulations.

The usage of typical constraints in the screening of boronic acid derivative libraries could lead to a false negative presentation with regard to binding poses and scoring function values, failing to replicate possible covalent bonds among boron and the amino acids Ser/Thr existing in the active site. Thus, alternate steps must be taken into account when we are dealing with hit compounds bearing boron in their structures producing plausible bindng poses. In particular, using standard hydrogen bond (HB) or contact constraints for Thr209 provided HA155 solutions with 100% off poses based only on the inhibitor’s shape score with minimum HB network scores. Alternatively, the introduction of custom constraints incorporating a smart pattern-like option is needed in order to better simulate the original HA155 query in our training sets (see Scheme 1). Additionally, smart patterns **I** and **II** reproduce a number of binding modes with other neighboring residues bearing an oxygen atom, such as Asp171/Tyr306 (and excepting Thr209). The statistics for our study groups are presented in Table 1.

**Figure 1:**
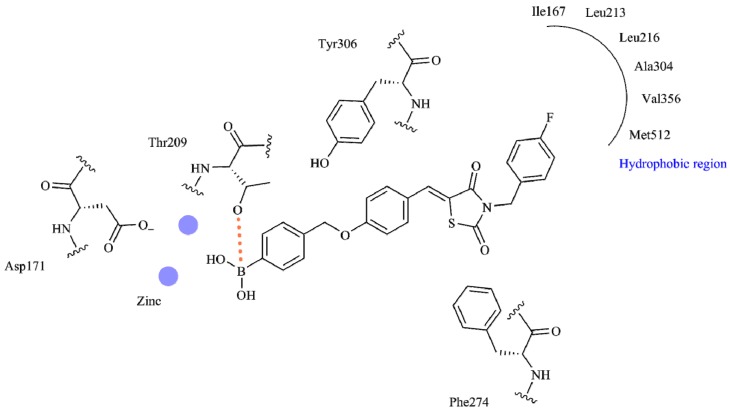
Schematic representation of the covalent bond as orange dashed lines between compound HA155 and neighboring residues of the autotaxin (ATX) active site.

Training set_1 used custom constraint **I** and provided the worst results. On the other hand, Training set_7 (with no active constraints) resulted in impressively high scoring functions, but entirely lacked the correct binding mode. Training set_3 had as active constraint patterns **I**, **II**, and **III**, and provided improved results. For the study of the remaining cases, all of the above-mentioned constraints (**I**–**IV**) were used (differentiated on the included distance). The distance was presented as a sphere with descending radius centered on the Thr209 hydroxyl group varying from preset 3.5 to 1.25 Angstroms (Å). The presented results were acceptable and could be used in screening as an active negative filter for boron compounds. Among Training sets_2, _4, _5, and _6 (based on their statistics), the most enriched proved to be Training set_4, with a cut-off radius of 2.5 Å. Figure 1 provides a detailed 2D map analysis of the ATX active site.

**Figure 2:**
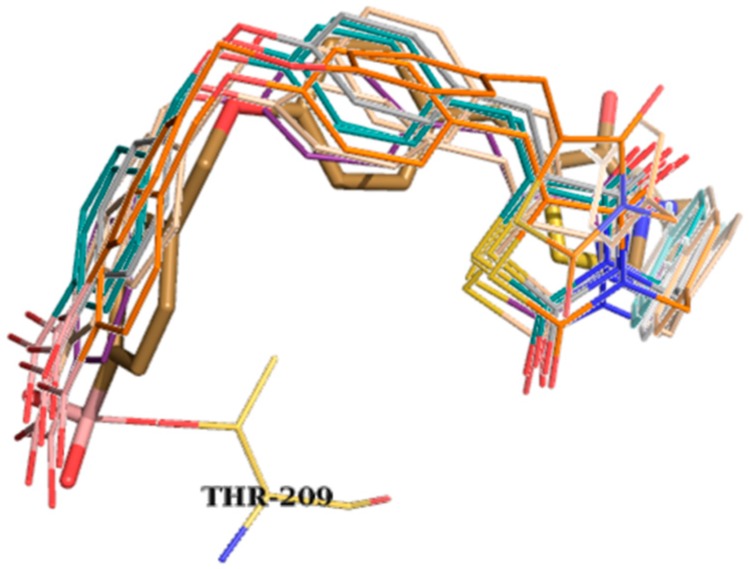

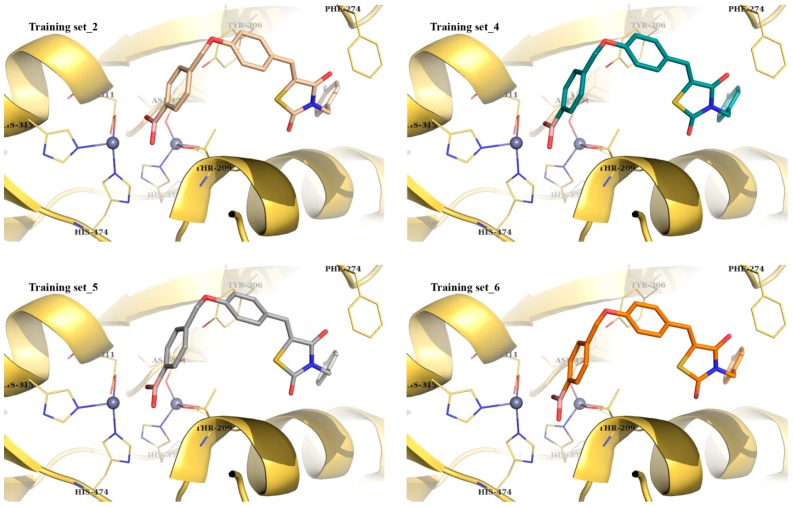
Overlay representation of query compound HA155 as gold sticks with Thr209 residue present and the predicted solutions as colored lines (**top**); best scoring poses of modeled compound HA155 within the active site of ATX (**bottom**). The figure was obtained by PyMol version 1.4.1 [30].

Images of the successful case studies are given in Figure 2, both in overlay with the original query compound HA155 and in detailed 3D representations inside the protein’s active site.

**Figure 3:**
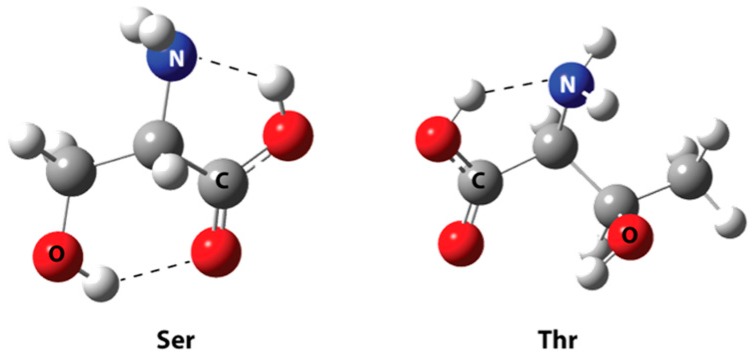
Optimized geometries of Serine and Threonine amino acids at B3LYP/6-31+G(d,p) level of theory with their respective intramolecular HBs as dashed lines.

The optimized geometries of serine and threonine amino acids are the global minima (as shown in Figure 3). Two hydrogen bonds are observed in serine. The first is located between the carboxylic hydrogen and the nitrogen of the backbone amine (1.929 Å), and the second HB is between the carboxylic oxygen and the residual hydroxyl group (2.040 Å). In threonine, the HBs can be observed between the carboxylic hydrogen and the amine group (1.899 Å).

**Figure 4:**
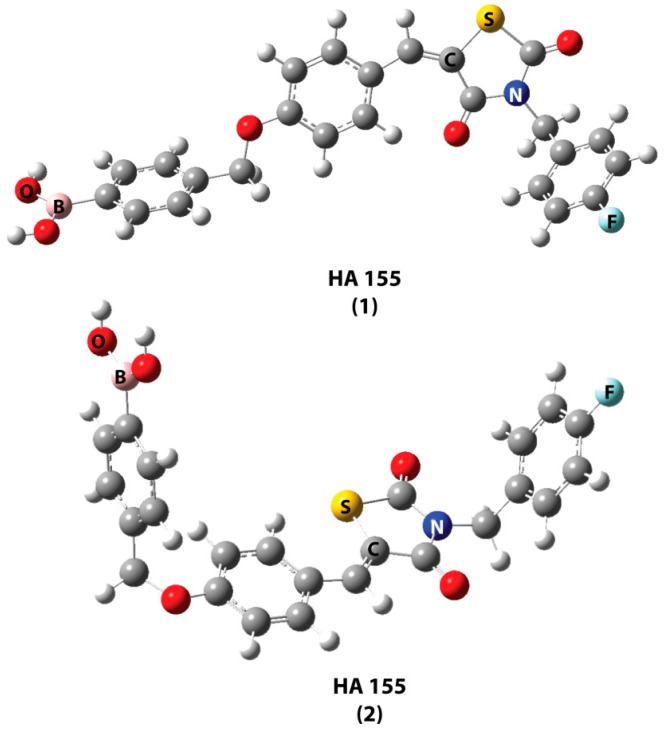
Optimized geometries of inhibitor HA155, a local minima conformer (**1**) and a global minima crystal structure conformer (**2**), at the B3LYP/6-31+G(d,p) level of theory.

The optimized geometries of the inhibitor HA155 are depicted in Figure 4. The HA155 (**2**) structure is the optimized crystal structure as experimentally isolated. This conformation of the inhibitor is more stable than the HA155 (**1**) structure, which is another local minimum conformation in the potential energy surface, varying by 2.02 kcal/mol, including the zero point correction energy.

**Figure 5:**
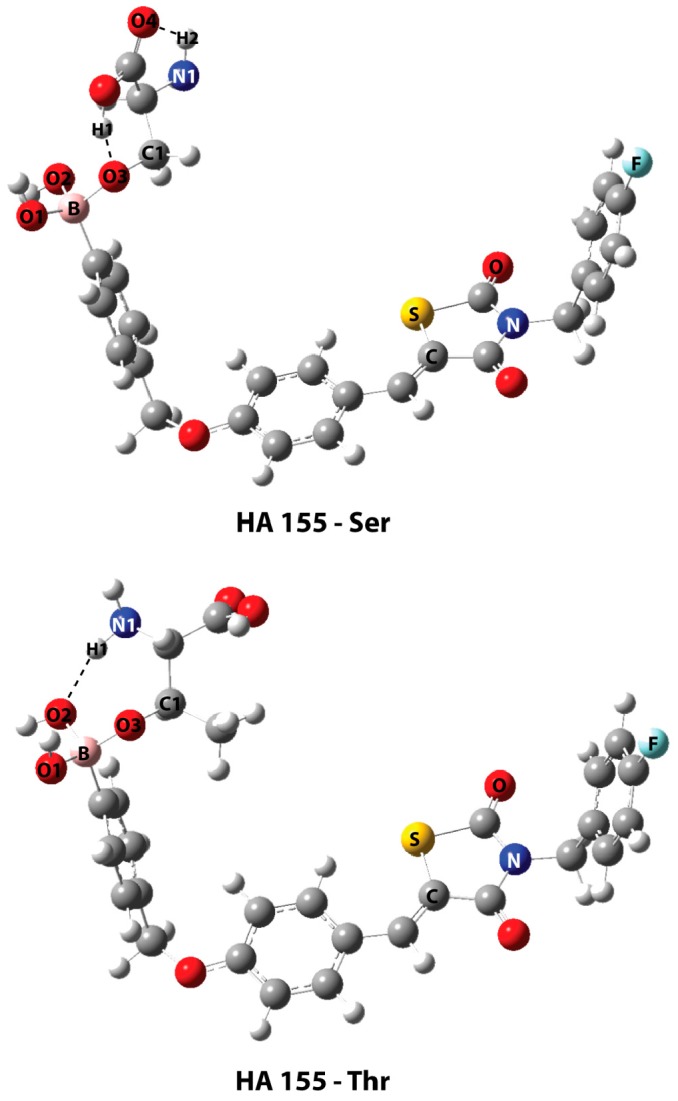
Optimized geometries of the bonded structures of the inhibitor HA155 with serine (**top**) and threonine (**bottom**) at the B3LYP/6-31+G(d,p) level of theory, with their respective intramolecular HBs as dashed lines.

**Figure 6:**
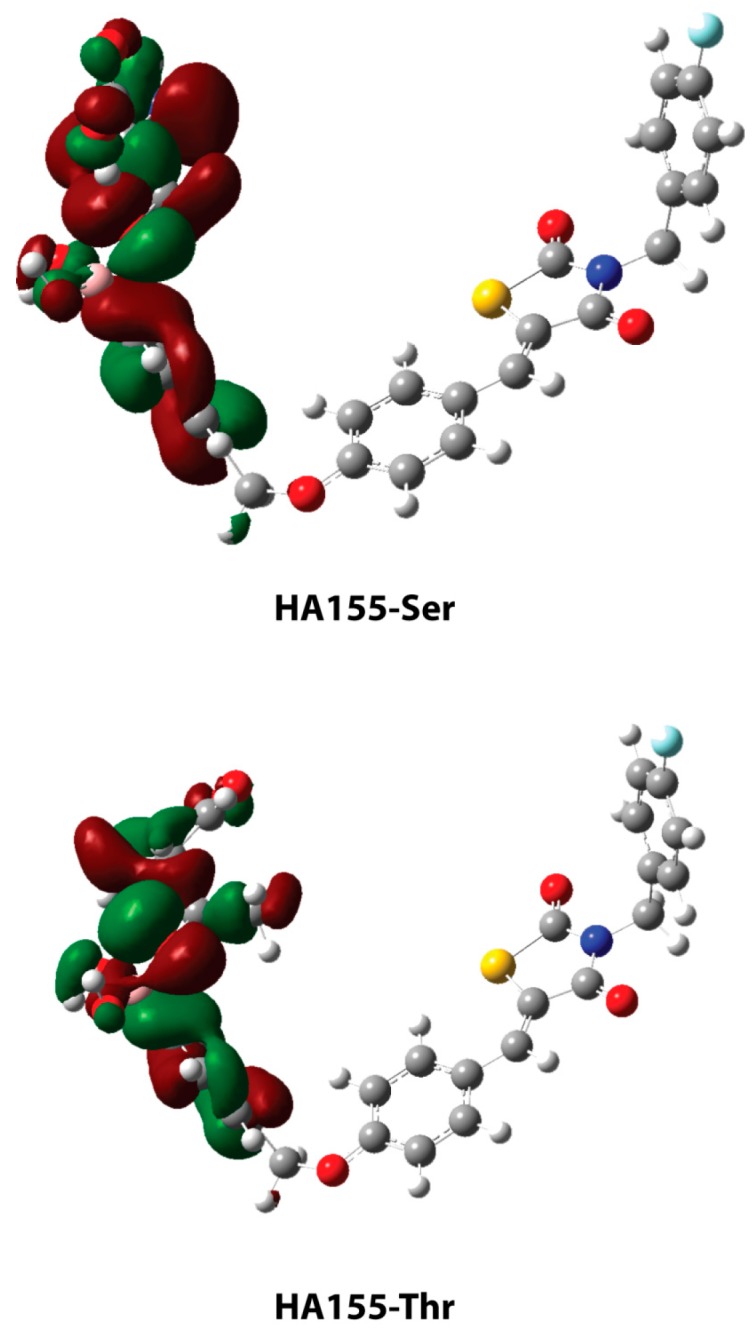
Highest occupied molecular orbitals (HOMOs) of the HA155-**Ser** (**top**) and HA155-**Thr** (**bottom**) at the B3LYP/6-31+G(d,p) level of theory.

The optimized geometries of the bonded serine and threonine with boronic acid (HA155) are presented in Figure 5. In both structures, the hydroxyl groups of the amino acids are bonded with the inhibitor’s boron atom, converting its hybridization to sp^3^. The geometric parameters are shown in Table 2. The highest occupied molecular orbitals (HOMO) of the two complexes are shown in Figure 6, depicting the electron density concentrated between the boron atom of compound HA155 and the amino acids. The natural bond orbital (NBO) calculations—both in HA155-**Ser** and HA155-**Thr** complexes—showed that the bond between boron and oxygen (B-O3, Figure 5) more correctly refers to a polar covalent bond. Based on these calculations, it seems that the occupation number in oxygen is approximately 1.65 electrons, and in boron 0.40 electrons, respectively. Accordingly, the degree of polarity for our models was calculated as 1.770 for HA155-**Thr** and 1.821 for HA155-**Ser**, with a threshold of 1.700 for the covalent bond.

The binding energies of the HA155-**Ser** complex were calculated both in the gas phase as well as in water solution, providing 311.18 and 300.13 kcal/mol, respectively, whereas the energies for HA155-**Thr** were 326.49 and 309.52 kcal/mol. The high binding energies found here support the formation of a weak covalent bond between compound HA155 and both Ser/Thr residues. These results provided extra evidence of the reversible nature of this bond, as it was also previously experimentally documented [13,15].

## 3. Computational Details

*Docking studies.* The target enzyme ATX (PDB entry 2XRG) co-crystallized with the small molecule inhibitor HA155 was used in our study [25]. All simulations were reproduced on a typical desktop PC running a Windows 7 64-bit operating system (Dual Core Intel Pentium 3.2 GHz CPU processors, RAM 8 GB), using OEDocking suite v. 3.0.1 programs (OpenEye Scientific Software, Inc., Santa Fe, NM, USA; www.eyesopen.com) [31,32,33] using Exhaustive Search Algorithm. Visualization of the docking solutions was performed with PyMol v. 1.4.1 software [30].

The PDB data were used to evaluate our results, and the HA155 molecule was represented in smile format compiled in a simple *.txt file, where with the use of Open Babel [34] we transformed it to the respective *.smi file. Consequently, we acquired its conformer library file with the use of Omega v.2.5.1.4 software (OpenEye Scientific Software, Inc., Santa Fe, NM, USA; www.eyesopen.com) [35,36] in *.oeb.gz format. Preparation of the respective *.pdb-formatted protein was done using the OEDocking v. 3.0.1 suite program MAKE RECEPTOR (OpenEye Scientific Software, Inc., Santa Fe, NM, USA; www.eyesopen.com) [31,32,33]. All water molecules were removed. The docking box was centered on the protein active site which included the co-crystallized HA155 molecule implementing a balanced site-shape potential by an outer contour docking space. No residue modifications were presented unless otherwise specified. Several different constraint options were utilized for residue Thr209 in an effort to better reproduce the original data, and the protein was then saved as a *.oeb-formatted file for further use. Command line-based docking was then performed, specifying the above prepared files as input for the OEDocking v. 3.0.1 suite software (OpenEye Scientific Software, Inc., Santa Fe, NM, USA; www.eyesopen.com) [31,32,33], generating 60 final poses for each model. All of the results were further refined by the use of OEDocking suite program FRED rescore v. 3.0.1 (OpenEye Scientific Software, Inc., Santa Fe, NM, USA; www.eyesopen.com) [31,32,33], providing the sorting of poses using default parameters. Scoring functions were based on Chemgauss4 values, and the solutions were saved as *.sdf files, offering the possibility of being used further for visualization purposes.

*Density Functional Theory (DFT) calculations.* All geometry optimization calculations were carried out on a typical desktop PC running a Windows 7 64-bit operating system (Intel i7 2.9 GHz CPU processors, RAM 4 GB), in gas phase, using the G09W [37] software package. The hybrid DFT method with Becke’s [38] three-parameter functional and the nonlocal correlation provided by the Lee, Yang, and Parr expression [39] (B3LYP) was used for optimization, employing the 6-31+G(d,p) basis set [40,41,42]. Frequency calculations for all optimized geometries ensured that the calculated structures were the global minima of the potential energy surface of the molecules. Single point calculations starting from the optimized geometry of all structures were also carried out in water solvent using the PCM [43,44,45] model as implemented in G09W software. Natural bond orbital analysis was performed using NBO 3.1 software package as implemented in the G09W package [37], in order to evaluate the covalent bond nature between the studied compounds.

## 4. Conclusions

Virtual screening of large libraries of boronic acid derivatives fail to dock in a natural mode, since they commonly form covalent bonds with Ser and/or Thr residues when they are presented in the protein active site. Hence, they are left out as false negatives both in regard to their binding poses and their scoring function values. This issue demands programs that can incorporate custom constraints such as smart pattern options or pinpoint selection.

Based on NBO calculations (performed originally herein), the formed bond between Ser/Thr residues is characterized more accurately as a polar covalent bond instead of a simple nonpolar covalent one. The presented results are acceptable, and could be used in screening as an active negative filter for boron compounds. The hydroxyl groups of amino acids are bonded with the inhibitor’s boron atom, converting its hybridization to sp^3^. Furthermore, the extremely high binding energies of the HA155-**Ser**/**Thr** complexes revealed that this reaction is not spontaneous. These theoretical data are also in accordance with the experimentally witnessed reversible binding of the inhibitor HA155 inside the ATX catalytic domain active site.

The findings described above highlight general options that need to be considered when large libraries of boron compounds are virtually screened to identify novel hits in drug design.

## Figures and Tables

**Table 1:** Summary statistics for our study groups.

Best Poses	General Score (%)	HBs Formed (%)	Chemgauss4 Score
^#^ Training set_1	68–93	87–95	−5.64
Training set_2	40–46	82–90	−9.48
Training set_3	20	94	−8.67
Training set_4	77–82	92–98	−9.35
Training set_5	82–85	71–95	−9.21
Training set_6	66–92	92–93	−9.05
* Training set_7	7–30	55–80	−12.18 (off position)

^#^ Smart pattern constraint **I**; * no active constraints. HB: hydrogen bond.

**Table 2:** Geometrical parameters of the HA155-**Ser** and HA155-**Thr**. The bonds are given in Å and the angles in degrees.

	HA155-**Ser**	HA155-**Thr**
*Bonds* *		
B-O1	1.460	1.465
B-O2	1.484	1.496
B-O3	1.521	1.495
*Angles*		
B-O3-C1	119.6	122.8
*Intermolecular Hydrogen Bond*		
H1…O3	1.568	-
H2…O4	2.210	-
H1…O2	-	1.955

* The atom numbering follows the same format as represented in Figure 4.

## Abbreviations

The following abbreviations are used in this manuscripts

2D	two-dimensional
3D	three-dimensional
ATX	autotaxin
B3LYP	Becke three-parameter Lee-Yang-Par
CPU	central processing unit
DFT	density functional theory
HB	hydrogen bond
HOMO	highest occupied molecular orbital
HTS	high-throughput screening
LPA	lysophosphatidic acid
LPC	lysophosphatidylcholine
NBO	natural bond orbital
PDB	protein data bank
PDE	phosphodiesterase
PPI	protein-protein interaction
RAM	random access memory
QSAR	quantitative structure-activity relationship
XRD	X-ray diffraction

## References

[1] Ballatore C., Huryn D.M., Smith A.B. (2013). Carboxylic acid (bio)isosteres in drug design. ChemMedChem.

[2] Adams J., Behnke M., Chen S., Cruickshank A.A., Dick L.R., Grenier L., Klunder J.M., Ma Y.T., Plamondon L., Stein R.L. (1998). Potent and selective inhibitors of the proteasome: Dipeptidyl boronic acids. Bioorg. Med. Chem. Lett..

[3] Lesnikowski Z.J. (2016). Recent developments with boron as a platform for novel drug design. Expert Opin. Drug. Discov..

[4] Dembitsky V.M., Smoum R., Al-Quntar A.A., Abu Ali H., Pergament I., Srebnik M. (2002). Natural occurrence of boron-containing compounds in plants, algae and microorganisms. Plant Sci..

[5] Trippier P.C., McGuigan C. (2010). Boronic acids in medicinal chemistry: Anticancer, antibacterial and antiviral applications. MedChemComm.

[6] Das B.C., Thapa P., Karki R., Schinke C., Das S., Kambhampati S., Banerjee S.K., Van Veldhuizen P., Verma A., Weiss L.M. (2013). Boron chemicals in diagnosis and therapeutics. Future Med. Chem..

[7] Jimenez-Aligaga K., Bermejo-Bescos P., Martin-Aragon S., Csaky A.G. (2013). Discovery of alkenylboronic acids as neuroprotective agents affecting multiple biological targets involved in Alzheimer’s disease. Bioorg. Med. Chem. Lett..

[8] Rock F.L., Mao W., Yaremchuk A., Tukalo M., Crepin T., Zhou H., Zhang Y.K., Hernandez V., Akama T., Baker S.J. (2007). An antifungal agent inhibits an aminoacyl-tRNA synthetase by trapping tRNA in the editing site. Science.

[9] Albers H.M., Ovaa H. (2012). Chemical evolution of autotaxin inhibitors. Chem. Rev..

[10] Albers H.M., Hendrickx L.J., van Tol R.J., Hausmann J., Perrakis A., Ovaa H. (2011). Structure-based design of novel boronic acid-based inhibitors of autotaxin. J. Med. Chem..

[11] Albers H.M., Dong A., van Meeteren L.A., Egan D.A., Sunkara M., van Tilburg E.W., Schuurman K., van Tellingen O., Morris A.J., Smyth S.S. (2010). Boronic acid-based inhibitor of autotaxin reveals rapid turnover of LPA in the circulation. Proc. Natl. Acad. Sci. USA.

[12] Albers H.M., van Meeteren L.A., Egan D.A., van Tilburg E.W., Moolenaar W.H., Ovaa H. (2010). Discovery and optimization of boronic acid based inhibitors of autotaxin. J. Med. Chem..

[13] Akama T., Baker S.J., Zhang Y.K., Hernandez V., Zhou H., Sanders V., Freund Y., Kimura R., Maples K.R., Plattner J.J. (2009). Discovery and structure-activity study of a novel benzoxaborole anti-inflammatory agent (AN2728) for the potential topical treatment of psoriasis and atopic dermatitis. Bioorg. Med. Chem. Lett..

[14] Minkkila A., Saario S.M., Kasnanen H., Leppanen J., Poso A., Nevalainen T. (2008). Discovery of boronic acids as novel and potent inhibitors of fatty acid amide hydrolase. J. Med. Chem..

[15] Knott K., Fishovitz J., Thorpe S.B., Lee I., Santos W.L. (2010). *N*-Terminal peptidic boronic acids selectively inhibit human ClpXP. Org. Biomol. Chem..

[16] Jacobs R.T., Nare B., Wring S.A., Orr M.D., Chen D., Sligar J.M., Jenks M.X., Noe R.A., Bowling T.S., Mercer L.T. (2011). SCYX-7158, an orally-active benzoxaborole for the treatment of stage 2 human African trypanosomiasis. PLoS Negl. Trop. Dis..

[17] Garcia-Soria G., Gonzalez-Galvez G., Argoud G.M., Gerstman M., Littlejohn T.W., Schwartz S.L., O’Farrell A.M., Li X., Cherrington J.M., Bennett C. (2008). The dipeptidyl peptidase-4 inhibitor PHX1149 improves blood glucose control in patients with type 2 diabetes mellitus. Diabetes Obes. Metab..

[18] Ban H.S., Usui T., Nabeyama W., Morita H., Fukuzawa K., Nakamura H. (2009). Discovery of boron-conjugated 4-anilinoquinazoline as a prolonged inhibitor of EGFR tyrosine kinase. Org. Biomol. Chem..

[19] Bandyopadhyay A., McCarthy K.A., Kelly M.A., Gao J. (2015). Targeting bacteria via iminoboronate chemistry of amine-presenting lipids. Nat. Commun..

[20] Mendes R.E., Alley M.R., Sader H.S., Biedenbach D.J., Jones R.N. (2013). Potency and spectrum of activity of AN3365, a novel boron-containing protein synthesis inhibitor, tested against clinical isolates of Enterobacteriaceae and nonfermentative Gram-negative bacilli. Antimicrob. Agents Chemother..

[21] Ramkumar K., Tambov K.V., Gundla R., Manaev A.V., Yarovenko V., Traven V.F., Neamati N. (2008). Discovery of 3-acetyl-4-hydroxy-2-pyranone derivatives and their difluoridoborate complexes as a novel class of HIV-1 integrase inhibitors. Bioorg. Med. Chem..

[22] Hansen M.M., Jolly R.A., Linder R.J. (2015). Boronic Acids and Derivatives-Probing the Structure-Activity Relationships for Mutagenicity. Org. Process Res. Dev..

[23] Katsamakas S., Bermperoglou E., Hadjipavlou-Litina D. (2015). Considering Autotaxin Inhibitors in Terms of 2D-QSAR and 3D-Mapping- Review and Evaluation. Curr. Med. Chem..

[24] Katsamakas S., Hadjipavlou-Litina D. (2013). Boronic Acid Based Inhibitors of Autotaxin: Understanding their Biological Role in Terms of Quantitative Structure Activity Relationships (QSAR). Lett. Drug Des. Discov..

[25] Hausmann J., Kamtekar S., Christodoulou E., Day J.E., Wu T., Fulkerson Z., Albers H.M., van Meeteren L.A., Houben A.J., van Zeijl L. (2011). Structural basis of substrate discrimination and integrin binding by autotaxin. Nat. Struct. Mol. Biol..

[26] Tsilikounas E., Kettner C.A., Bachovchin W.W. (1992). Identification of serine and histidine adducts in complexes of trypsin and trypsinogen with peptide and nonpeptide boronic acid inhibitors by proton NMR spectroscopy. Biochemistry.

[27] Adeniyi A.A., Muthusamy R., Soliman M.E. (2016). New drug design with covalent modifiers. Expert Opin. Drug Discov..

[28] Mah R., Thomas J.R., Shafer C.M. (2014). Drug discovery considerations in the development of covalent inhibitors. Bioorg. Med. Chem. Lett..

[29] Smith M.C., Gestwicki J.E. (2012). Features of protein-protein interactions that translate into potent inhibitors: Topology, surface area and affinity. Expert Rev. Mol. Med..

[30] The PyMOL Molecular Graphics System.

[31] McGann M. (2011). FRED pose prediction and virtual screening accuracy. J. Chem. Inf. Model..

[32] McGaughey G.B., Sheridan R.P., Bayly C.I., Culberson J.C., Kreatsoulas C., Lindsley S., Maiorov V., Truchon J.F., Cornell W.D. (2007). Comparison of topological, shape, and docking methods in virtual screening. J. Chem. Inf. Model..

[33] McGann M.R., Almond H.R., Nicholls A., Grant J.A., Brown F.K. (2003). Gaussian docking functions. Biopolymers.

[34] O’Boyle N.M., Banck M., James C.A., Morley C., Vandermeersch T., Hutchison G.R. (2011). Open Babel: An open chemical toolbox. J. Cheminform..

[35] Hawkins P.C., Nicholls A. (2012). Conformer generation with OMEGA: Learning from the data set and the analysis of failures. J. Chem. Inf. Model..

[36] Hawkins P.C., Skillman A.G., Warren G.L., Ellingson B.A., Stahl M.T. (2010). Conformer generation with OMEGA: Algorithm and validation using high quality structures from the Protein Databank and Cambridge Structural Database. J. Chem. Inf. Model..

[37] Frisch M.J., Trucks G.W., Schlegel H.B., Scuseria G.E., Robb M.A., Cheeseman J.R., Scalmani G., Barone V., Mennucci B., Petersson G.A. (2009). Gaussian 09, Revision A. 02.

[38] Becke A.D. (1993). Density-functional thermochemistry. III. The role of exact exchange. J. Chem. Phys..

[39] Lee C.T., Yang W.T., Parr R.G. (1988). Development of the Colle-Salvetti Correlation-Energy Formula into a Functional of the Electron-Density. Phys. Rev. B.

[40] Clark T., Chandrasekhar J., Spitznagel G.W., Schleyer P.V. (1983). Efficient Diffuse Function-Augmented Basis Sets for Anion Calculations. III. The 3-21+G Basis Set for First-Row Elements, Li-F. J. Comput. Chem..

[41] Krishnan R., Binkley J.S., Seeger R., Pople J.A. (1980). Self-consistent molecular orbital methods. XX. A basis set for correlated wave functions. J. Chem. Phys..

[42] McLean A.D., Chandler G.S. (1980). Contracted Gaussian basis sets for molecular calculations. I. Second row atoms, Z=11–18. J. Chem. Phys..

[43] Amovilli C., Barone V., Cammi R., Cancès E., Cossi M., Mennucci B., Pomelli C.S., Tomasi J. (1998). Recent Advances in the Description of Solvent Effects with the Polarizable Continuum Model. Advances in Quantum Chemistry.

[44] Tomasi J., Persico M. (1994). Molecular-Interactions in Solution—An Overview of Methods Based on Continuous Distributions of the Solvent. Chem. Rev..

[45] Miertuš S., Scrocco E., Tomasi J. (1981). Electrostatic interaction of a solute with a continuum. A direct utilizaion of AB initio molecular potentials for the prevision of solvent effects. Chem. Phys..

